# Maternal and Perinatal Outcomes Associated with Maternal HIV Infection in a Tertiary Hospital in Eastern Cape Province, South Africa

**DOI:** 10.3390/idr18040081

**Published:** 2026-07-30

**Authors:** Viwe Sodo-Mbotya, Ntandazo Dlatu, Geoffrey A. B. Buga

**Affiliations:** 1 Department of Obstetrics and Gynaecology, Faculty of Health Sciences, iYunivesithi Walter Sisulu, Nelson Mandela Drive, Mthatha 5100, South Africa; geoffreybuga@msn.com; 2 Walter Sisulu Institute for Clinical Governance and Healthcare Administration, School of Public Health, Faculty of Health Sciences, iYunivesithi Walter Sisulu, Mthatha 5100, South Africa; ndlatu@wsu.ac.za

**Keywords:** HIV, maternal outcomes, perinatal outcomes, ART, PMTCT, low birthweight, neonatal mortality, antenatal care, Eastern Cape, South Africa

## Abstract

**Background**: Despite substantial progress in prevention of mother-to-child transmission (PMTCT) programmes and widespread access to antiretroviral therapy (ART), maternal HIV infection remains associated with adverse maternal and neonatal outcomes in many high HIV-burden settings. This study compared maternal and perinatal outcomes among women living with HIV and HIV-negative women delivering at a tertiary referral hospital in the Eastern Cape Province, South Africa. **Methods**: A retrospective comparative cohort study was conducted using routinely collected clinical records of 600 women (300 HIV-positive and 300 HIV-negative) who delivered at Nelson Mandela Academic Hospital between January and December 2019. Maternal, obstetric, and neonatal characteristics were compared according to maternal HIV status. Associations were evaluated using chi-square tests, multivariable logistic regression, Kaplan–Meier survival analysis, and Cox proportional hazards regression models. **Results**: Women living with HIV were older, had higher parity, and were more likely to have documented anaemia and delayed antenatal care attendance than HIV-negative women. HIV-exposed pregnancies had higher frequencies of preterm birth (26.3% vs. 20.3%) and low birthweight (LBW). In adjusted analyses, maternal HIV-positive status remained independently associated with increased odds of LBW (AOR = 1.88; 95% CI: 1.18–3.00; *p* = 0.008). LBW was independently associated with neonatal intensive care unit (NICU) admission (AOR = 2.45; 95% CI: 1.46–4.11; *p* < 0.001) and an increased hazard of in-hospital neonatal mortality (HR = 2.40; 95% CI: 1.55–3.70; *p* < 0.001). Maternal HIV-positive status (HR = 1.75; 95% CI: 1.12–2.71; *p* = 0.015) and unsuppressed maternal viral load (HR = 2.05; 95% CI: 1.13–3.73; *p* = 0.018) were also associated with increased hazards of neonatal mortality. However, these findings should be interpreted cautiously, given the limited number of neonatal mortality events (*n* = 32). Among HIV-exposed infants with documented HIV test results, the observed mother-to-child transmission rate was 1.7%. However, incomplete infant follow-up and HIV testing data limited the precision of this estimate. Among women living with HIV, birthweight did not differ significantly according to the timing of ART initiation. **Conclusions**: In this tertiary referral hospital cohort, maternal HIV infection was associated with adverse maternal and neonatal outcomes, particularly anemia, preterm birth, and LBW. LBW emerged as an important predictor of neonatal morbidity and mortality. These findings support continued efforts to strengthen integrated HIV and maternal healthcare services, promote early antenatal care engagement, maintain maternal viral suppression, and improve monitoring and care of high-risk neonates. Given the retrospective observational design, incomplete follow-up for selected outcomes, limited numbers of neonatal mortality events, and the tertiary referral setting, the findings should be interpreted as associations rather than evidence of causal relationships and may not be generalizable to lower-level healthcare facilities or community-based obstetric populations.

## 1. Introduction

Human immunodeficiency virus (HIV) remains one of the most important global public health challenges, with sub-Saharan Africa continuing to bear a disproportionate share of the epidemic. South Africa has the largest HIV treatment programme globally. It is home to an estimated 7.8 million people living with HIV, with women of reproductive age remaining disproportionately affected by the disease [1]. Consequently, HIV infection continues to have important implications for maternal, fetal, and neonatal health outcomes.

The Eastern Cape Province is among the South African provinces most affected by HIV, with antenatal HIV prevalence estimates exceeding 30% in recent years [2,3]. Nelson Mandela Academic Hospital (NMAH), a tertiary referral hospital serving predominantly rural and socioeconomically disadvantaged communities in the O.R. Tambo District, provides a relevant setting in which to examine maternal and neonatal outcomes among women living with HIV. The province continues to experience substantial socioeconomic and healthcare challenges, including poverty, transportation barriers, workforce shortages, and delayed access to maternal healthcare services, all of which may influence pregnancy outcomes [4,5]. The scale-up of antiretroviral therapy (ART) and implementation of prevention of mother-to-child transmission (PMTCT) programmes have transformed HIV care and substantially reduced vertical HIV transmission. In the absence of intervention, mother-to-child transmission rates may range from 15% to 45%; however, widespread ART coverage and PMTCT implementation have reduced transmission rates to below 3.5% in South Africa [4]. These achievements have been further strengthened by World Health Organization (WHO) recommendations supporting universal lifelong ART for all pregnant and breastfeeding women living with HIV, irrespective of CD4 cell count or clinical stage [5]. Despite these advances, maternal HIV infection remains associated with adverse maternal and perinatal outcomes in many settings. Studies from sub-Saharan Africa have reported higher frequencies of preterm birth, low birthweight (LBW), stillbirth, and neonatal morbidity among women living with HIV and their infants compared with HIV-negative populations [6,7,8,9,10]. A systematic review from the region reported significantly increased odds of LBW and preterm delivery among women living with HIV [6]. Similarly, studies conducted in South Africa and neighboring countries have documented elevated risks of adverse birth outcomes, including preterm delivery, fetal growth restriction, and stillbirth [7,8]. Neonates born to women living with HIV have also been reported to experience higher risks of prematurity, impaired growth, hospitalization, and mortality during the neonatal period [9,10]. The relationship between maternal HIV infection and adverse pregnancy outcomes is complex and likely reflects interactions among biological, clinical, behavioral, and socioeconomic factors. Maternal viral suppression, timing of ART initiation, treatment adherence, nutritional status, comorbid conditions, quality of antenatal care, and broader social determinants of health may all influence maternal and neonatal outcomes [11,12,13,14,15]. Furthermore, findings remain inconsistent across studies, particularly in the contemporary ART era, where widespread treatment availability has altered the clinical course of HIV infection and improved maternal health outcomes [11,12,13,14,15]. These inconsistencies highlight the need for context-specific evidence from high HIV-burden settings. Although several studies have examined pregnancy outcomes among women living with HIV in South Africa, relatively limited evidence is available from rural and underserved areas of the Eastern Cape Province, particularly within tertiary referral settings managing complex pregnancies. In addition, data on maternal and neonatal outcomes during the period of mature ART and PMTCT programme implementation remain limited [15,16,17,18]. Understanding these outcomes is important for evaluating ongoing maternal and child health challenges and identifying opportunities to strengthen integrated HIV and obstetric care services. Therefore, this study aimed to compare maternal and perinatal outcomes among women living with HIV and HIV-negative women delivering at a tertiary referral hospital in the Eastern Cape Province, South Africa. By generating locally relevant evidence, the study sought to examine associations between maternal HIV status and pregnancy outcomes in a high HIV-burden setting and to inform efforts to strengthen integrated maternal HIV services, antenatal care, PMTCT programmes, and neonatal healthcare.

## 2. Methods

### 2.1. Study Design and Setting

This retrospective comparative cohort study evaluated maternal and perinatal outcomes among women living with HIV and HIV-negative women who delivered at Nelson Mandela Academic Hospital (NMAH), Eastern Cape Province, South Africa. Participants were classified according to maternal HIV status, and maternal and neonatal outcomes were compared between the two exposure groups using routinely collected clinical records. NMAH is a tertiary referral hospital located in the O.R. Tambo District Municipality, Eastern Cape Province, South Africa. The hospital serves as the main referral centre for district and regional hospitals across a predominantly rural catchment area characterized by substantial socioeconomic deprivation, limited healthcare resources, and barriers to accessing healthcare services. The study included eligible deliveries that occurred between 1 January and 31 December 2019.

During the study period, South Africa had fully implemented the World Health Organization (WHO) recommendation of universal lifelong antiretroviral therapy (ART) (Option B+) for all pregnant and breastfeeding women living with HIV, irrespective of CD4 cell count or clinical stage. Routine antenatal care included HIV counselling and testing, viral load monitoring, ART initiation and follow-up, and prevention of mother-to-child transmission (PMTCT) services. The year 2019 was selected because it represents a mature phase of national HIV program implementation, characterized by widespread ART availability and well-established PMTCT services. Because NMAH is a tertiary referral hospital, the study population may have included a higher proportion of high-risk pregnancies than is typically seen in primary healthcare or community-based settings. Consequently, the findings should be interpreted in the context of a tertiary referral population.

### 2.2. Study Population and Data Sources

The study population comprised women who delivered at NMAH between 1 January and 31 December 2019. A total of 600 maternal records were included, consisting of 300 women living with HIV and 300 HIV-negative women. As this was a retrospective study based on routinely collected clinical data, no formal a priori sample size calculation was undertaken. The final sample size was determined by the number of eligible women living with HIV identified during the study period, together with an equal number of HIV-negative women selected from the same source population for comparison. Eligible participants had documented maternal HIV status and complete information on the primary maternal and neonatal outcomes. Women with undocumented HIV status, indeterminate HIV test results, or missing primary outcome data were excluded.

HIV-negative women were selected systematically from the same delivery population using labour ward delivery registers as the sampling frame until the required sample size was achieved. No individual or frequency matching was performed. Data were abstracted from maternity case records, antenatal clinic cards, labour ward registers, and neonatal records. Information obtained from multiple sources was cross-checked, where available, to improve data completeness and accuracy. Data abstraction was performed using a structured, pre-tested data collection form with predefined variable definitions and standardized coding procedures.

### 2.3. Study Variables

Data were collected across four domains: maternal demographic characteristics, obstetric history, clinical characteristics, and neonatal outcomes. Maternal demographic variables included maternal age, parity, and antenatal care (ANC) booking status. ANC booking was categorized as early (≤20 weeks’ gestation) or late (>20 weeks’ gestation). Clinical variables included hypertensive disorders of pregnancy (HDP), anemia, diabetes mellitus, maternal infections, timing of antiretroviral therapy (ART) initiation, maternal viral load, and CD4 cell count, when available. Women living with HIV were categorized based on whether ART had been initiated before or during pregnancy.

Obstetric variables included gestational age at delivery and mode of delivery. Gestational age was determined from the last menstrual period and/or obstetric ultrasound findings, where documented.

Neonatal outcomes included birthweight, Apgar scores at one and five minutes, neonatal intensive care unit (NICU) admission, stillbirth, infant HIV test results, and in-hospital neonatal mortality. Low birthweight (LBW) was defined as a birthweight <2500 g, while preterm birth was defined as delivery before 37 completed weeks of gestation.

For multivariable analyses examining maternal morbidity, antenatal complications were analysed as a composite outcome comprising one or more documented antenatal conditions, including hypertensive disorders of pregnancy, anaemia, diabetes mellitus, and maternal infections. This composite outcome was used because several individual complications occurred infrequently, thereby limiting statistical power for separate multivariable analyses. Nevertheless, these conditions represent distinct clinical entities with different biological mechanisms and potentially different associations with maternal HIV infection. Therefore, findings regarding the composite antenatal complication outcome should be interpreted with caution and not extrapolated to individual complications.

### 2.4. Missing Data

Data completeness was assessed before statistical analysis through systematic review of all study variables. Most demographic, obstetric, and neonatal variables were well documented, with minimal missing data. However, maternal viral load measurements were unavailable for approximately 22.3% of women living with HIV because of incomplete routine laboratory monitoring or documentation. Similarly, infant HIV testing and follow-up data were incomplete for some HIV-exposed infants, limiting analyses related to early infant HIV outcomes and vertical HIV transmission.

Descriptive analyses were performed using all available observations for each variable, whereas multivariable logistic regression and Cox proportional hazards models were based on complete-case analyses. Multiple imputation was not performed because the retrospective nature of the study limited the ability to determine the mechanisms underlying missingness and to justify the assumptions required for valid imputation. Consequently, some multivariable analyses were based on reduced sample sizes, which may have decreased statistical precision. The potential influence of missing data on the study findings is discussed in the Discussion and Limitations sections.

### 2.5. Statistical Analysis

Statistical analyses were performed using IBM SPSS Statistics for Windows, Version 27.0 (IBM Corp., Armonk, NY, USA). Before analysis, the dataset was examined for completeness, consistency, coding errors, implausible values, and outliers. Categorical variables were summarized using frequencies and percentages, whereas continuous variables were summarized using means with standard deviations (SDs) or medians with interquartile ranges (IQRs), as appropriate. Data distribution was assessed using graphical methods, including histograms and normal probability plots, together with summary statistics.

Comparisons between women living with HIV and HIV-negative women were performed using Pearson’s chi-square test or Fisher’s exact test for categorical variables and independent-samples *t*-tests or Mann–Whitney U tests for continuous variables, depending on data distribution. Among women living with HIV, mean birthweight according to timing of ART initiation (before pregnancy versus during pregnancy) was compared using an independent-samples *t*-test. Multivariable logistic regression models were constructed to identify factors independently associated with adverse maternal and neonatal outcomes. Variables with *p* < 0.20 in univariable analyses, together with clinically relevant covariates identified a priori, were considered for inclusion in multivariable models. Maternal age and parity were retained in all models because of their established clinical importance. Candidate variables included maternal HIV status, maternal age, parity, ANC booking status, hypertensive disorders of pregnancy, anaemia, diabetes mellitus, gestational age at delivery, and other clinically relevant maternal characteristics. Model calibration was evaluated using the Hosmer–Lemeshow goodness-of-fit test, while multicollinearity was assessed using variance inflation factors (VIFs). No evidence of problematic multicollinearity was identified. Results are reported as adjusted odds ratios (AORs) with 95% confidence intervals (CIs).

Survival analyses were undertaken to evaluate factors associated with in-hospital neonatal mortality. Time-to-event was defined as the interval between birth and in-hospital neonatal death. Neonates discharged alive were censored on the date of hospital discharge. Deaths occurring after discharge were unavailable and were therefore not included in the survival analyses. Kaplan–Meier methods were used to estimate neonatal survival according to maternal HIV status, and survival distributions were compared using the log-rank test.

Multivariable Cox proportional hazards regression models were fitted to estimate adjusted hazard ratios (HRs) and 95% confidence intervals. Covariates included maternal HIV status, birthweight, gestational age, ANC booking status, maternal complications, and other clinically relevant variables. The proportional hazards assumption was assessed using graphical inspection of log-minus-log survival plots and Schoenfeld residuals and was found to be adequately satisfied. Given the relatively small number of neonatal mortality events, survival analyses should be considered exploratory, and the corresponding hazard ratio estimates should be interpreted with appropriate caution. All statistical tests were two-sided, and *p*-values < 0.05 were considered statistically significant.

## 3. Result

### 3.1. Maternal Demographic Characteristics

A total of 600 maternity records were included in the analysis, comprising 300 women living with HIV and 300 HIV-negative women. Women living with HIV were significantly older than HIV-negative women, with a mean age of 34 ± 6 years compared with 30 ± 4 years, respectively (t = 9.72, *p* < 0.001). The distribution of age categories differed significantly between the groups (χ^2^ = 70.71, *p* < 0.001), with a substantially greater proportion of women living with HIV being older than 35 years, whereas younger women (<20 years) were more frequently represented among HIV-negative participants. Parity also differed significantly according to maternal HIV status (χ^2^ = 25.99, *p* < 0.001). Nulliparity was more common among HIV-negative women, while higher parity was observed more frequently among women living with HIV. Overall, women living with HIV were older and had a greater cumulative reproductive history than HIV-negative women. Detailed maternal demographic characteristics according to HIV status are presented in Table 1.

### 3.2. Maternal Clinical Characteristics

Maternal clinical characteristics according to HIV status are presented in Table 2. Women living with human immunodeficiency virus (HIV) had significantly higher frequencies of hypertensive disorders of pregnancy (HDP) and anaemia than HIV-negative women. In contrast, previous caesarean section and diabetes mellitus were more frequently documented among HIV-negative women. Maternal infections and other clinical conditions were uncommon in both groups and did not differ significantly according to maternal HIV status.

Antenatal care (ANC) utilization also differed significantly between the groups. Late ANC booking, defined as initiation of antenatal care after 20 weeks’ gestation, was more common among women living with HIV than among HIV-negative women. Overall, women living with HIV experienced a less favourable clinical profile, characterized by higher frequencies of HDP, anaemia, and delayed ANC attendance. Detailed maternal clinical characteristics are presented in Table 2.

### 3.3. Neonatal Outcomes

Neonatal outcomes according to maternal HIV status are presented in Table 3. Infants born to women living with human immunodeficiency virus (HIV) had significantly lower mean birthweight and shorter gestational age at delivery than infants born to HIV-negative women. Adverse birth outcomes, including preterm birth, low Apgar scores at five minutes, stillbirth, and neonatal anaemia, were more frequently observed among HIV-exposed infants.

In contrast, the crude proportion of in-hospital neonatal mortality did not differ significantly according to maternal HIV status. Because neonatal mortality was subsequently evaluated using time-to-event methods that accounted for the timing of events and censoring, adjusted survival analyses are presented separately in Section 3.8 and Section 3.9. Overall, HIV exposure was associated with less favourable neonatal outcomes, particularly reduced birthweight, shorter gestation, and higher frequencies of selected adverse neonatal conditions.

### 3.4. Birthweight According to Timing of ART Initiation

Among women living with human immunodeficiency virus (HIV), the mean birthweight was lower among infants born to women who initiated antiretroviral therapy (ART) before pregnancy than among those who initiated ART during pregnancy (Table 4). Specifically, the mean birthweight was 2415 ± 385 g among infants whose mothers initiated ART before pregnancy (n = 205), compared with 2512 ± 340 g among those whose mothers initiated ART during pregnancy (n = 77). However, this difference did not reach statistical significance (independent-samples *t*-test: t = 1.69, *p* = 0.060). These findings suggest that, within this cohort, the timing of ART initiation was not significantly associated with neonatal birthweight. Given the observational nature of the study and the relatively small number of women who initiated ART during pregnancy, these findings should be interpreted cautiously. Further studies with larger sample sizes and more detailed information on ART exposure, treatment adherence, and viral suppression are needed to better understand the relationship between the timing of ART initiation and neonatal birth outcomes.

### 3.5. Predictors of Antenatal Complications

Antenatal complications were analyzed as a composite outcome defined by the presence of one or more documented maternal complications, including hypertensive disorders of pregnancy (HDP), anaemia, diabetes mellitus, and maternal infections. Multivariable logistic regression analysis was performed to identify factors independently associated with this composite outcome (Table 5). After adjustment for maternal age, parity, antenatal care (ANC) booking status, timing of antiretroviral therapy (ART) initiation, and maternal viral load status, women living with human immunodeficiency virus (HIV) had lower odds of the composite antenatal complication outcome than HIV-negative women (adjusted odds ratio [AOR] = 0.35; 95% confidence interval [CI]: 0.22–0.55; *p* < 0.001). In contrast, initiation of ART before pregnancy was associated with higher odds of the composite outcome compared with ART initiation during pregnancy (AOR = 1.68; 95% CI: 1.02–2.76; *p* = 0.041). Neither unsuppressed maternal viral load (AOR = 1.94; 95% CI: 0.91–4.12; *p* = 0.085) nor maternal age greater than 35 years (AOR = 1.42; 95% CI: 0.90–2.25; *p* = 0.127) was significantly associated with the composite outcome after adjustment. Notably, the direction of the adjusted association for HIV status differed from the descriptive findings presented in Table 2, where higher frequencies of specific complications, particularly HDP and anaemia, were observed among women living with HIV. This apparent discrepancy may reflect the influence of covariate adjustment, residual confounding, model specification, or the heterogeneous nature of the composite outcome. Because the outcome combined several clinically distinct conditions with potentially different relationships to maternal HIV infection, the adjusted association should be interpreted as relating to the composite outcome as a whole rather than to any individual antenatal complication. Consequently, these findings should be interpreted cautiously.

### 3.6. Predictors of Low Birthweight

Multivariable logistic regression analysis was performed to identify factors independently associated with low birthweight (LBW) (Table 6). The model included maternal age, parity, antenatal care (ANC) booking status, hypertensive disorders of pregnancy, anaemia, gestational age at delivery, maternal human immunodeficiency virus (HIV) status, timing of antiretroviral therapy (ART) initiation, and maternal viral load status. After adjustment for potential confounders, maternal HIV-positive status was independently associated with increased odds of LBW compared with HIV-negative status (adjusted odds ratio [AOR] = 1.88; 95% confidence interval [CI]: 1.18–3.00; *p* = 0.008). This finding was consistent with the descriptive analyses, which demonstrated lower mean birthweight among infants born to women living with HIV. Maternal age greater than 35 years was not significantly associated with LBW after adjustment (AOR = 1.31; 95% CI: 0.84–2.05; *p* = 0.230). Similarly, ART initiation before pregnancy (AOR = 1.12; 95% CI: 0.71–1.78; *p* = 0.628) and unsuppressed maternal viral load (AOR = 1.47; 95% CI: 0.79–2.75; *p* = 0.220) were not significantly associated with LBW in the adjusted model.

Overall, maternal HIV-positive status emerged as the only significant independent predictor of LBW in this analysis.

### 3.7. Predictors of Neonatal Intensive Care Unit Admission

Multivariable logistic regression analysis was performed to identify factors independently associated with admission to the neonatal intensive care unit (NICU) (Table 7). The model included gestational age at delivery, maternal HIV status, maternal age, parity, antenatal care booking status, maternal complications, timing of ART initiation, and other clinically relevant covariates. Low birthweight was independently associated with increased odds of NICU admission (AOR = 2.45; 95% CI: 1.46–4.11; *p* < 0.001). Infants with LBW had approximately 2.5 times greater odds of requiring NICU admission than infants with birthweights of 2500 g or greater. Maternal HIV-positive status was not significantly associated with NICU admission after adjustment for covariates (AOR = 1.11; 95% CI: 0.68–1.82; *p* = 0.674). Likewise, initiation of ART before pregnancy was not significantly associated with NICU admission (AOR = 1.36; 95% CI: 0.77–2.42; *p* = 0.292). These findings suggest that LBW, rather than maternal HIV status or timing of ART initiation, was the principal factor associated with NICU admission in this cohort.

### 3.8. Survival Analysis of In-Hospital Neonatal Mortality

Multivariable Cox proportional hazards regression analysis was performed to identify factors associated with in-hospital neonatal mortality (Table 8). The model included gestational age at delivery, birthweight, antenatal care (ANC) booking status, maternal complications, maternal human immunodeficiency virus (HIV) status, timing of antiretroviral therapy (ART) initiation, maternal viral load status, and other clinically relevant covariates. Although the crude proportion of in-hospital neonatal mortality did not differ significantly between infants born to HIV-positive and HIV-negative women (5.7% vs. 5.0%; *p* = 0.660), maternal HIV-positive status was associated with an increased hazard of in-hospital neonatal mortality in the adjusted model (hazard ratio [HR] = 1.75; 95% confidence interval [CI]: 1.12–2.71; *p* = 0.015). Low birthweight was independently associated with increased hazard of in-hospital neonatal mortality (HR = 2.40; 95% CI: 1.55–3.70; *p* < 0.001), indicating that infants with birthweights below 2500 g experienced substantially higher mortality risk during hospitalization. Similarly, unsuppressed maternal viral load was associated with increased hazard of neonatal mortality compared with suppressed viral load (HR = 2.05; 95% CI: 1.13–3.73; *p* = 0.018). In contrast, initiation of ART before pregnancy was not significantly associated with in-hospital neonatal mortality after adjustment (HR = 1.38; 95% CI: 0.87–2.20; *p* = 0.172). Overall, maternal HIV-positive status, low birthweight, and unsuppressed maternal viral load were independently associated with increased hazards of in-hospital neonatal mortality in this cohort. However, because only 32 neonatal deaths were observed during follow-up, these findings should be interpreted cautiously. The hazard ratios represent adjusted associations and should not be interpreted as evidence of causal relationships.

### 3.9. Kaplan–Meier Analysis of In-Hospital Neonatal Survival

**Figure 1:**
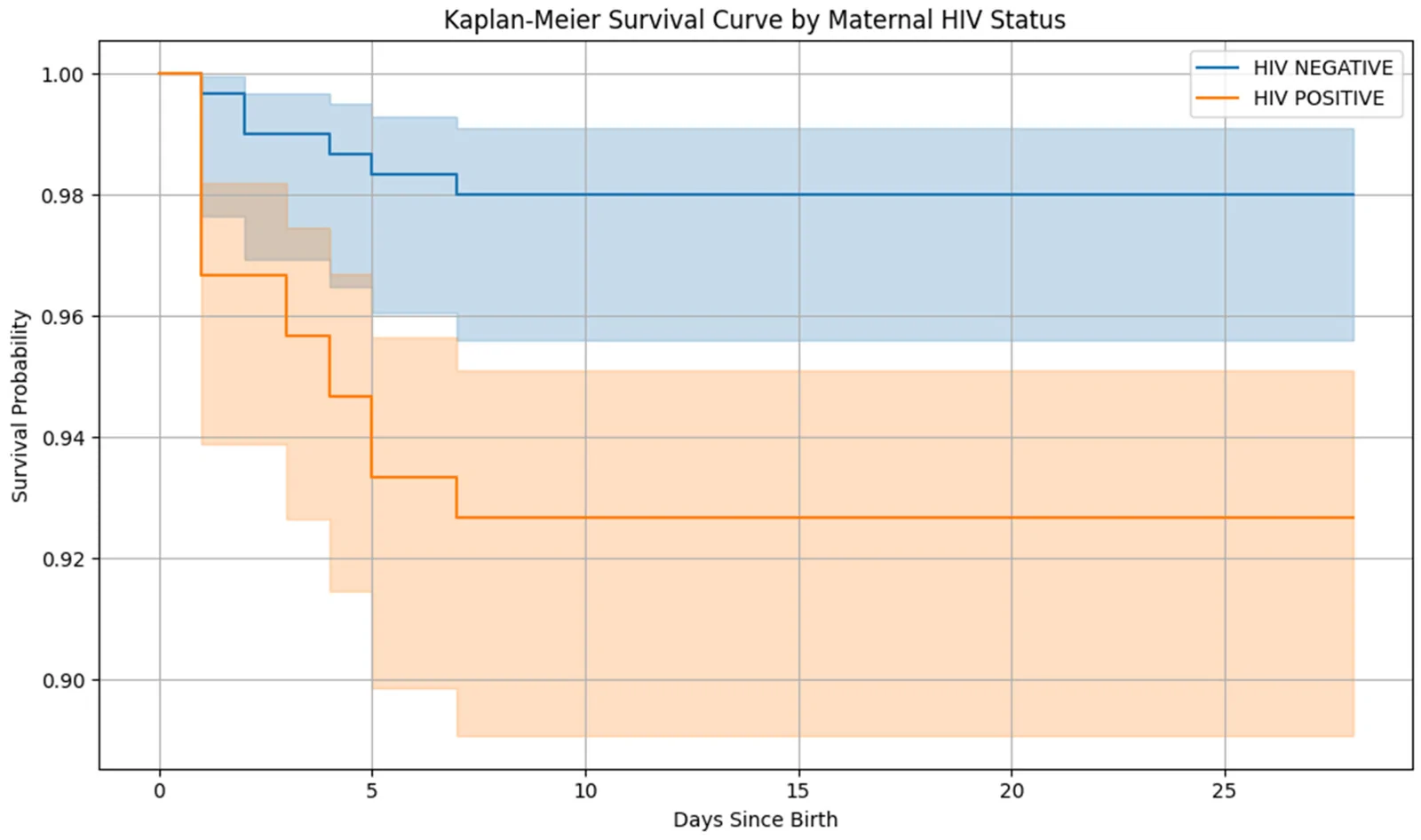
Kaplan–Meier curves for in-hospital neonatal survival according to maternal HIV status.

Kaplan–Meier survival analysis was performed to evaluate in-hospital neonatal survival according to maternal human immunodeficiency virus (HIV) status (Figure 1). Infants born to women living with HIV demonstrated lower estimated survival probabilities during hospitalization than infants born to HIV-negative women. The survival curves began to separate during the early postnatal period, suggesting that most in-hospital neonatal deaths occurred within the first few days after birth. A total of 17 in-hospital neonatal deaths occurred among infants born to women living with HIV, compared with 15 deaths among infants born to HIV-negative women. Although the crude proportions of neonatal mortality did not differ significantly between the groups (Table 3), Kaplan–Meier analysis accounts for both event timing and censoring, thereby providing a more comprehensive assessment of survival throughout the hospitalization period. The visual separation of the survival curves was consistent with the findings of the adjusted Cox proportional hazards model presented in Table 8, which identified maternal HIV-positive status as being associated with an increased hazard of in-hospital neonatal mortality. However, given the relatively small number of mortality events observed during follow-up, the survival estimates should be interpreted with caution and treated as exploratory.

Kaplan–Meier survival curves showing the probability of in-hospital neonatal survival among infants born to HIV-positive and HIV-negative women. Time-to-event was defined as the interval between birth and in-hospital neonatal death. Neonates discharged alive were censored at hospital discharge. Shaded regions represent 95% confidence intervals, and tick marks indicate censored observations. A total of 17 in-hospital neonatal deaths occurred among infants born to women living with HIV, compared with 15 deaths among infants born to HIV-negative women. The number-at-risk table beneath the figure displays the number of neonates remaining under observation at each time point. The figure is presented for descriptive purposes and should be interpreted alongside the adjusted Cox proportional hazards model results shown in Table 8.

## 4. Discussion

This study evaluated maternal and perinatal outcomes among women living with HIV and HIV-negative women delivering at a tertiary referral hospital in the Eastern Cape Province, South Africa. Despite widespread access to antiretroviral therapy (ART) and established prevention of mother-to-child transmission (PMTCT) programmes, maternal HIV-positive status remained associated with several adverse maternal and neonatal outcomes. Women living with HIV experienced higher frequencies of anaemia, hypertensive disorders of pregnancy, and delayed antenatal care (ANC) booking, while HIV-exposed infants had lower birthweight, shorter gestational age, higher rates of preterm birth, lower Apgar scores, and higher stillbirth frequencies. In adjusted analyses, maternal HIV-positive status remained associated with low birthweight, while low birthweight and unsuppressed maternal viral load were associated with adverse neonatal outcomes. These findings suggest that important maternal and neonatal health disparities persist despite substantial progress in HIV care and PMTCT services [19,20,21,22].

The demographic profile of the study population showed that women living with HIV were significantly older and had higher parity than HIV-negative women. These findings are consistent with national and regional epidemiological evidence demonstrating a higher HIV burden among older women of reproductive age in Southern Africa [19,20,21,22]. The higher parity observed among women living with HIV may reflect broader reproductive patterns in the era of effective ART. However, the observational design precludes conclusions regarding the underlying reasons for these differences. Because maternal age and parity are established determinants of pregnancy outcomes, these variables were included as adjustment factors in the multivariable analyses.

Women living with HIV experienced higher frequencies of anaemia and hypertensive disorders of pregnancy than HIV-negative women. The elevated prevalence of anaemia among women living with HIV is consistent with previous studies reporting associations between HIV infection and adverse haematological outcomes during pregnancy [23,24]. This relationship may reflect the combined influence of chronic inflammation, nutritional deficiencies, coexisting infections, and treatment-related factors. Similarly, the higher frequency of hypertensive disorders observed among women living with HIV is consistent with evidence suggesting complex interactions between HIV infection, ART exposure, maternal immune function, and underlying cardiovascular risk factors [25].

An important finding was the apparent discrepancy between the descriptive analyses and the multivariable model evaluating antenatal complications. Although women living with HIV experienced higher frequencies of specific complications, particularly anaemia and hypertensive disorders of pregnancy, HIV-positive status was inversely associated with the composite antenatal complication outcome after adjustment. This finding should be interpreted with considerable caution. The observed association may reflect the influence of covariate adjustment, residual confounding, model specification, overadjustment, or the heterogeneous nature of the composite outcome, which combines clinically distinct conditions, including anemia, hypertensive disorders of pregnancy, diabetes mellitus, and maternal infections. These conditions may have different biological pathways and differing relationships with maternal HIV status. The use of a composite outcome was intended to improve statistical efficiency given the limited frequency of some individual complications; however, this approach may obscure condition-specific associations. Consequently, the adjusted association should not be interpreted as evidence that HIV infection is protective against antenatal complications. Rather, it highlights the complexity of the relationship between maternal HIV status and pregnancy morbidity and underscores the need for future studies to evaluate individual antenatal complications separately [26,27].

Antenatal care utilization was suboptimal among women living with HIV, with more than one-third initiating ANC after 20 weeks’ gestation. Delayed ANC attendance has been associated with missed opportunities for early risk identification, timely ART optimization, and management of pregnancy-related complications [28,29]. These findings may reflect persistent structural barriers to healthcare access, including transportation challenges, socioeconomic constraints, and healthcare system limitations that continue to affect maternal health services in rural and underserved settings.

Neonatal outcomes differed according to maternal HIV status. HIV-exposed infants had lower mean birthweight, shorter gestational age, and higher frequencies of preterm birth, low Apgar scores, stillbirth, and neonatal anaemia. These findings are consistent with studies from South Africa and other high HIV-burden settings that have reported adverse neonatal outcomes among infants born to women living with HIV [30,31,32,33]. Although the mechanisms underlying these associations were not directly evaluated in this study, they are likely multifactorial. They may involve maternal health status, placental function, inflammation, viral suppression, treatment-related factors, and broader social determinants of health. Low birthweight emerged as the strongest predictor of neonatal intensive care unit (NICU) admission and was independently associated with in-hospital neonatal mortality. These findings are consistent with extensive evidence identifying low birthweight as a major determinant of neonatal morbidity and mortality globally [34,35]. Importantly, the association between low birthweight and NICU admission persisted after adjustment for gestational age and other covariates, highlighting the substantial vulnerability of low-birthweight infants within this population.

The survival analysis demonstrated that maternal HIV-positive status, low birthweight, and unsuppressed maternal viral load were associated with increased hazards of in-hospital neonatal mortality. Although crude mortality proportions did not differ significantly between HIV-positive and HIV-negative women, the adjusted Cox proportional hazards model identified significant associations after accounting for follow-up time and measured covariates. These findings should be interpreted as observational associations rather than evidence of causal effects. Residual confounding remains possible because important factors such as maternal nutritional status, socioeconomic conditions, ART adherence, maternal disease severity, and quality of antenatal care were not consistently available in the clinical records. The survival analysis should also be interpreted cautiously because only 32 in-hospital neonatal deaths were observed during follow-up. The relatively small number of outcome events may have reduced the precision and stability of the hazard ratio estimates and increased the potential for model overfitting. Consequently, these findings should be regarded as exploratory and hypothesis-generating rather than definitive evidence of independent prognostic effects. Larger prospective studies are needed to confirm the observed associations and provide more robust estimates of risk.

Although the observed mother-to-child transmission rate was low (1.7%), reflecting the success of PMTCT programmes, incomplete infant follow-up and HIV testing data limited the precision of this estimate. Continued strengthening of maternal viral load monitoring, ART adherence support, and infant follow-up services remains essential to sustain gains achieved in PMTCT programmes. Overall, the findings emphasize the continued importance of integrated HIV and maternal healthcare services. While ART and PMTCT programmes have substantially improved maternal and neonatal health outcomes, additional efforts are required to strengthen early ANC engagement, maintain maternal viral suppression, improve fetal growth monitoring, and enhance care for preterm and low-birthweight infants in high HIV-burden settings.

### 4.1. Strengths and Limitations

This study included a relatively large cohort of women delivering in a high HIV-burden setting. It evaluated both maternal and neonatal outcomes, allowing a comprehensive assessment of outcomes associated with maternal HIV status. The inclusion of multivariable logistic regression and survival analyses enabled adjustment for important confounders and evaluation of time-to-event outcomes. Furthermore, the use of routinely collected clinical data provides valuable real-world evidence from a tertiary referral setting where complex maternal and neonatal conditions are frequently encountered. Several limitations should be acknowledged. First, the retrospective design relied on routinely collected clinical records and was therefore subject to incomplete documentation, information bias, and missing data. Missing viral load data and incomplete infant HIV testing reduced the completeness of some analyses and may have affected the precision of selected estimates. Second, the study was conducted at a single tertiary referral hospital. Consequently, the findings may not be directly generalizable to primary healthcare facilities, district hospitals, or community-based obstetric populations. The referral nature of the hospital may also have introduced referral bias because women with high-risk pregnancies and obstetric complications are more likely to be referred for specialist care. Third, residual confounding may persist despite statistical adjustment. Information on maternal nutritional status, educational attainment, household income, socioeconomic status, ART adherence, duration of ART exposure, maternal disease severity, and quality of antenatal care was not consistently available. It therefore could not be fully accounted for in the analyses. An additional limitation relates to the survival analysis. Although Cox proportional hazards modelling was used to account for differences in follow-up time and censoring, only 32 neonatal mortality events occurred during the study period. This relatively small number of events may have limited statistical power, increased the possibility of model overfitting, and reduced the precision of hazard ratio estimates. Finally, the observational nature of the study precludes causal conclusions. Therefore, all reported findings should be interpreted as associations rather than evidence of causal relationships.

### 4.2. Recommendations

The findings of this study suggest several priorities for clinical practice and public health programmes. Strategies to promote earlier ANC engagement should be strengthened, particularly among women living with HIV. Community outreach initiatives, health education programs, and interventions that address barriers to healthcare access may improve timely attendance at ANC. The association between unsuppressed maternal viral load and increased neonatal mortality underscores the need to strengthen viral load monitoring, adherence support, and timely clinical management for women with detectable viral loads during pregnancy. Maintaining maternal viral suppression should remain a central component of HIV care. Given the observed associations between maternal HIV-positive status, low birthweight, and adverse neonatal outcomes, enhanced fetal growth monitoring, nutritional support, and early identification of high-risk pregnancies should be prioritized. Strengthening neonatal care services and improving management of preterm and low-birthweight infants may further improve neonatal outcomes. Routine screening and management of anaemia and hypertensive disorders of pregnancy should also be reinforced, particularly among women living with HIV. Future prospective studies incorporating detailed information on ART exposure, adherence, viral suppression, maternal disease severity, socioeconomic factors, and quality of antenatal care are needed. Future research should also evaluate individual antenatal complications separately rather than relying solely on composite outcome measures, as different complications may have distinct relationships with maternal HIV status.

## 5. Conclusions

Maternal HIV-positive status was associated with several adverse maternal and neonatal outcomes, including anaemia, preterm birth, low birthweight, and higher hazards of in-hospital neonatal mortality within this tertiary referral cohort. Despite substantial progress achieved through ART and PMTCT programmes, important maternal and neonatal health disparities remain in high HIV-burden settings. The findings support continued efforts to strengthen early ANC engagement, sustain maternal viral suppression, improve fetal growth monitoring, and enhance neonatal care services for vulnerable infants. Integrated HIV and maternal healthcare services remain essential for improving maternal and neonatal health outcomes. Because this study was conducted in a tertiary referral hospital using retrospective observational data, the findings should be interpreted as associations rather than evidence of causal effects. They may not be directly generalizable to lower-level healthcare facilities or community-based obstetric populations.

## Figures and Tables

**Table 1:** Maternal demographic characteristics of study participants according to HIV status.

Variable	HIV-Negative (*n* = 300)	HIV-Positive (*n* = 300)	Test Statistic	*p*-Value
Maternal age (years), Mean ± SD	30 ± 4	34 ± 6	t = 9.72	<0.001
Age category, *n* (%)			χ^2^ = 70.71	<0.001
<20 years	58 (19.3)	4 (1.3)		
20–34 years	184 (61.3)	173 (57.7)		
≥35 years	58 (19.3)	123 (41.0)		
Parity, *n* (%)			χ^2^ = 25.99	<0.001
0	116 (38.7)	62 (20.7)		
1	66 (22.0)	73 (24.3)		
2	66 (22.0)	73 (24.3)		
3	36 (12.0)	64 (21.3)		
≥4	13 (4.3)	19 (6.3)		

Abbreviations: HIV, human immunodeficiency virus; SD, standard deviation. Footnote: Continuous variables were compared using independent-samples *t*-tests. Categorical variables were compared using Pearson’s chi-square tests. Percentages are presented within HIV-status groups.

**Table 2:** Maternal clinical characteristics of study participants according to HIV status.

Variable	HIV-Negative (*n* = 300), *n* (%)	HIV-Positive (*n* = 300), *n* (%)	Test Statistic	*p*-Value
HDP	149 (49.7)	198 (66.0)	χ^2^ = 14.86	<0.001
Anaemia	69 (23.0)	114 (38.0)	χ^2^ = 15.89	<0.001
Previous caesarean section	95 (31.7)	60 (20.0)	χ^2^ = 10.81	0.001
Diabetes mellitus	48 (16.0)	30 (10.0)	χ^2^ = 4.76	0.029
Maternal infections	5 (1.6)	8 (2.7)	Fisher’s exact test	0.402
Other clinical conditions	3 (1.0)	4 (1.3)	Fisher’s exact test	1.000
Late ANC booking (>20 weeks)	64 (21.3)	104 (34.7)	χ^2^ = 13.27	<0.001

Abbreviations: HIV, human immunodeficiency virus; HDP, hypertensive disorders of pregnancy; ANC, antenatal care; *n*, number of participants; χ^2^, Pearson’s chi-square statistic. Footnote: Data are presented as *n* (%). Pearson’s chi-square tests were used for categorical variables unless expected cell counts were less than five, in which case Fisher’s exact test was applied. Percentages are reported within HIV-status groups.

**Table 3:** Neonatal outcomes according to maternal HIV status.

Variable	HIV-Negative (*n* = 300)	HIV-Positive (*n* = 300)	Test Statistic	*p*-Value
Birthweight (kg), mean ± SD	2.92 ± 0.55	2.74 ± 0.61	t = 3.79	<0.001
Gestational age (weeks), mean ± SD	37.5 ± 2.4	36.1 ± 2.8	t = 6.57	<0.001
Preterm birth (<37 weeks)	61 (20.3)	79 (26.3)	χ^2^ = 4.02	0.045
Low Apgar score (<7 at five minutes)	28 (9.2)	44 (14.7)	χ^2^ = 4.62	0.032
Stillbirth	12 (4.0)	22 (7.3)	χ^2^ = 3.91	0.048
Neonatal anaemia	45 (15.0)	90 (30.0)	χ^2^ = 16.47	<0.001
In-hospital neonatal mortality	15 (5.0)	17 (5.7)	χ^2^ = 0.19	0.660

Abbreviations: HIV, human immunodeficiency virus; SD, standard deviation; *n*, number of participants; kg, kilograms; t, independent-samples *t*-test statistic; χ^2^, Pearson’s chi-square statistic. Footnote: Continuous variables were compared using independent-samples *t*-tests, while categorical variables were compared using Pearson’s chi-square tests. Percentages are presented within maternal HIV-status groups. In-hospital neonatal mortality refers to deaths occurring before hospital discharge. Time-to-event analyses evaluating neonatal survival are presented separately in Section 3.8 and Section 3.9.

**Table 4:** Birthweight according to timing of ART initiation among women living with HIV.

Timing of ART Initiation	*n*	Birthweight (g), Mean ± SD	Test Statistic	*p*-Value
ART initiated before pregnancy	205	2415 ± 385		
ART initiated during pregnancy	77	2512 ± 340	t = 1.69	0.060

Abbreviations: HIV, human immunodeficiency virus; ART, antiretroviral therapy; SD, standard deviation; g, grams; *n*, number of participants; t, independent-samples *t*-test statistic. Footnote: Mean birthweight was compared between women who initiated ART before pregnancy and those who initiated ART during pregnancy using an independent-samples *t*-test. No statistically significant difference in mean birthweight was observed between the groups (*p* = 0.060).

**Table 5:** Multivariable logistic regression model of factors associated with the composite antenatal complication outcome.

Variable	Reference Category	AOR	95% CI	*p*-Value
HIV-positive status	HIV-negative	0.35	0.22–0.55	<0.001
Maternal age > 35 years	≤35 years	1.42	0.90–2.25	0.127
ART initiated before pregnancy	ART initiated during pregnancy	1.68	1.02–2.76	0.041
Unsuppressed viral load	Suppressed viral load	1.94	0.91–4.12	0.085

Abbreviations: HIV, human immunodeficiency virus; ART, antiretroviral therapy; AOR, adjusted odds ratio; CI, confidence interval. Footnote: The composite antenatal complication outcome was defined as the occurrence of one or more documented antenatal complications, including hypertensive disorders of pregnancy, anaemia, diabetes mellitus, and maternal infections. The multivariable model was adjusted for maternal age, parity, antenatal care booking status, HIV status, timing of ART initiation, and maternal viral load status. HIV-negative status, maternal age ≤ 35 years, ART initiation during pregnancy, and suppressed viral load served as reference categories. Model diagnostics indicated no evidence of problematic multicollinearity among included variables.

**Table 6:** Multivariable logistic regression model of factors associated with low birthweight.

Variable	Reference Category	AOR	95% CI	*p*-Value
HIV-positive status	HIV-negative	1.88	1.18–3.00	0.008
Maternal age > 35 years	≤35 years	1.31	0.84–2.05	0.230
ART initiated before pregnancy	ART initiated during pregnancy	1.12	0.71–1.78	0.628
Unsuppressed viral load	Suppressed viral load	1.47	0.79–2.75	0.220

Abbreviations: HIV, human immunodeficiency virus; ART, antiretroviral therapy; AOR, adjusted odds ratio; CI, confidence interval; low birthweight. Footnote: The model was adjusted for maternal age, parity, antenatal care booking status, hypertensive disorders of pregnancy, anaemia, gestational age at delivery, maternal HIV status, timing of ART initiation, and maternal viral load status. HIV-negative status, maternal age ≤ 35 years, ART initiation during pregnancy, and suppressed viral load served as reference categories.

**Table 7:** Multivariable logistic regression model of factors associated with neonatal intensive care unit admission.

Variable	Reference Category	AOR	95% CI	*p*-Value
HIV-positive status	HIV-negative	1.11	0.68–1.82	0.674
ART initiated before pregnancy	ART initiated during pregnancy	1.36	0.77–2.42	0.292
Low birthweight (<2500 g)	Birthweight ≥ 2500 g	2.45	1.46–4.11	<0.001

Abbreviations: HIV, human immunodeficiency virus; ART, antiretroviral therapy; AOR, adjusted odds ratio; CI, confidence interval; neonatal intensive care unit; low birthweight. Footnote: The model was adjusted for gestational age at delivery, maternal age, parity, antenatal care booking status, maternal complications, maternal HIV status, timing of ART initiation, and other clinically relevant covariates. Low birthweight was defined as birthweight less than 2500 g. HIV-negative status, ART initiation during pregnancy, and birthweight ≥ 2500 g served as reference categories.

**Table 8:** Multivariable Cox proportional hazards model of factors associated with in-hospital neonatal mortality.

Variable	Reference Category	HR	95% CI	*p*-Value
HIV-positive status	HIV-negative	1.75	1.12–2.71	0.015
Low birthweight (<2500 g)	Birthweight ≥ 2500 g	2.40	1.55–3.70	<0.001
ART initiated before pregnancy	ART initiated during pregnancy	1.38	0.87–2.20	0.172
Unsuppressed viral load	Suppressed viral load	2.05	1.13–3.73	0.018

Abbreviations: HIV, human immunodeficiency virus; ART, antiretroviral therapy; HR, hazard ratio; CI, confidence interval; antenatal care. Footnote: Time-to-event was defined as the interval from birth to in-hospital neonatal death. Neonates discharged alive were censored at hospital discharge. The model was adjusted for gestational age at delivery, birthweight, antenatal care booking status, maternal complications, maternal HIV status, timing of ART initiation, maternal viral load status, and other clinically relevant covariates. HIV-negative status, birthweight ≥ 2500 g, ART initiation during pregnancy, and suppressed viral load served as reference categories. Assessment of the proportional hazard’s assumption using graphical methods and Schoenfeld residuals showed no substantial violations.

## Data Availability

The datasets generated and/or analysed during the current study are not publicly available because they contain confidential patient information and are subject to ethical and institutional restrictions. De-identified data may be made available from the corresponding author upon reasonable request and with permission from the Walter Sisulu University Human Research Ethics Committee and the Eastern Cape Department of Health, in accordance with applicable ethical and data protection regulations.

## Abbreviations

ANC	Antenatal Care
AOR	Adjusted Odds Ratio
ART	Antiretroviral Therapy
CD4	Cluster of Differentiation 4 T-Lymphocyte Count
CI	Confidence Interval
C/S	Caesarean Section
DM	Diabetes Mellitus
HDP	Hypertensive Disorders of Pregnancy
HIV	Human Immunodeficiency Virus
HR	Hazard Ratio
IQR	Interquartile Range
LBW	Low Birthweight
MTCT	Mother-to-Child Transmission
NICU	Neonatal Intensive Care Unit
NMAH	Nelson Mandela Academic Hospital
OR	Odds Ratio
O.R. Tambo	Oliver Reginald Tambo District Municipality
PMTCT	Prevention of Mother-to-Child Transmission
SD	Standard Deviation
SPSS	Statistical Package for the Social Sciences
VIF	Variance Inflation Factor
WHO	World Health Organization
